# The effect of infra low frequency–neurofeedback training on pistol shooting performance and attention in semi-skilled players

**DOI:** 10.3389/fnhum.2025.1487737

**Published:** 2025-01-22

**Authors:** Safoura Bakhtafrooz, Maryam Kavyani, Alireza Farsi, Saeed Alboghebeish

**Affiliations:** Department of Cognitive and Behavioral Science and Technology in Sport, Faculty of Sport Sciences and Health, Shahid Beheshti University, Tehran, Iran

**Keywords:** infra-low frequency neurofeedback, endogenous neuromodulation, shooting, attention network, sports performance, self-regulation

## Abstract

**Purpose:**

Neurofeedback (NF) typically involves an operant conditioning or other reinforcement protocol aimed at self-regulating patterns of brain activation. Endogenous Neuromodulation, characterized by the absence of discrete reinforcers, has emerged over the last two decades with the extension of training into the infra-low frequency regime, i.e., below 0.1 Hz. Specifically, Infra-Low Frequency (ILF) Neurofeedback training has demonstrated efficacy in enhancing the self-organization and regulation of the central nervous system in considerable generality. The present study explores a pivotal question: Can Infra-Low Frequency (ILF) Neurofeedback, acknowledged for its influence on arousal, vigilance, and emotional states, effectively enhance both attention generally and shooting performance specifically? Additionally, we explored whether the training exerted beneficial effects on three attentional networks—Conflict, Orienting, and Alerting.

**Methods:**

To assess shooting performance, we employed the Shooter’s Coordination Analysis Target Training (SCATT), while attention networks were gauged through the Attention Network Test (ANT). Twenty semi-skilled pistol shooters, aged 28–40, underwent both the ANT and SCATT assessments before and after completing 20 half-hour ILF-Neurofeedback sessions. The participants were randomly assigned to two groups: an ILF NFB group, which underwent 20 sessions of ILF NFB training, and a control group that received no NFB.

**Results:**

Our findings revealed that ILF-Neurofeedback significantly enhanced performance. In the ANT, the training led to a reduction in Conflict and an increase in Orienting and Alerting.

**Conclusion:**

The study demonstrates the effectiveness of ILF-Neurofeedback in improving shooting performance, and in positively impacting all three attention networks assessed by the ANT.

## 1 Introduction

Neurofeedback training (NFT) is a recognized self-regulation technique known for enhancing performance by targeting EEG frequency bands with reinforcement techniques. In the context of athletics, its effectiveness remains a subject of debate, even as the method is routinely used in Olympic and professional sports. While a systematic review by Mirifar et al. (2017) questioned its impact on sports performance (Mirifar et al., 2017), a meta-analysis by Xiang et al. (2018) demonstrated the potential of NFT in improving athletes’ performance (Xiang et al., 2018). These studies encompassed various EEG bands, such as Sensorimotor Rhythm (SMR), alpha, theta, and Slow Cortical Potentials (SCPs), significantly improving sports performance (Cheng et al., 2015; Faridnia et al., 2012; Gruzelier et al., 2014; Mikicin et al., 2015; Paul et al., 2012; Strizhkova et al., 2012).

A significant development in NFT was the exploration of Slow Cortical Potentials (SCP), which influence motor and cognitive preparation (Birbaumer et al., 1990). Clinical work led to the discovery of the Optimal Response Frequency (ORF) principle in the late nineties, which then led to the extension of training into the Infra-Low Frequency (ILF) regime, operating below 0.1 Hz, in the early 21st century (Othmer et al., 2013). While standard SCP training is event-focused, ILF training is frequency-based. This introduces unique technical challenges, as feedback must be provided on slowly varying signals. The targets are the dynamic connectivity relationships of our Resting State Networks, the Default Mode in particular. Consequently, signals are derived from bipolar montage. They track the differential surface potential, which is directly reflective of local cortical activation. The training impacts a number of physiological processes, including arousal, vigilance, emotional states, and autonomic balance, by targeting slow cortical potentials and modulating resting-state networks. Such methods are integral to self-regulation and have been related to cognitive and motor performance improvements through enhanced autonomic balance and emotional resilience (Grin-Yatsenko et al., 2020; Othmer, 2011; Othmer and Othmer, 2016).

However, despite its potential in addressing mood and emotional disorders, the effectiveness of ILF-Neurofeedback in enhancing sports performance remains relatively under-investigated, particularly in precision sports such as shooting. This prompts the primary question of our research: Can ILF Neurofeedback, known for its influence on arousal, vigilance, and emotional states (Othmer and Othmer, 2016), enhance attention and shooting performance?

Previous research in shooting has suggested that NFT has the potential to enhance sports performance. Hammond (2007) suggests that neurofeedback has the potential to improve shooting performance by quieting the mind. Paul et al. (2012) found that NFT improved psychological and electroencephalographic measures in shooters, leading to enhanced accuracy and performance (Paul et al., 2012). Rostami et al. (2012) demonstrated that NFT improved shooting performance (Rostami et al., 2012). However, the majority of research has focused on higher-frequency NFT.

Pistol Shooting, an activity characterized by precision elements (e.g., target accuracy), consistency (e.g., stability), and the ability to maintain steadfastness (continuity of state) (Brown et al., 2013), is recognized as a self-paced sport. In shooting, athletes must finely tune their cognitive abilities, including motivation (Salleh et al., 2020), working memory, attention, as well as physiological conditions (Ortega and Wang, 2018) such as blood pressure, heart rate, and respiration, to prepare themselves for the most challenging conditions. Li et al. (2021) showed that elite athletes in shooting performance had better interoceptive attention, which is the awareness of and focus on bodily signals, compared to non-elite athletes (Li et al., 2021). Lu et al. (2021) found that elite shooting athletes possessed more efficient attention networks, encompassing alerting, orienting, and conflict control (Lu et al., 2021). Additionally, Vrbik et al. (2021) demonstrated that an external focus of attention, oriented toward movement outcome, enhanced precision in recreational shooters.

To understand the role of attention in shooting more comprehensively, we turn to the Attention Network Test (ANT), which provides a framework for assessing attention. According to Posner and Petersen (1990), attention comprises three networks: alerting, orienting, and conflict control. The state of vigilance sustains the alerting network, which is crucial for athletes maintaining focus during shooting. The orienting network directs attention to specific sensory inputs or locations, aiding shooters in adapting to body signals and environmental factors. The conflict control network resolves interference, essential for coordinating body movements and minimizing distractions, especially in precision sports like shooting (Petersen and Posner, 2012).

The ANT, developed by Fan et al. (2002), combines cued reaction times and flanker tasks to measure these three attention networks (Fan et al., 2002). Participants react speedily to the orientation of a target arrow amidst distractor arrows, revealing differences in reaction times for alerting, orienting, and conflict control. The ANT’s reliability and convenience have led to its widespread adoption in various studies (Fan et al., 2005; Mannarelli et al., 2019; Rueda et al., 2004; Williams et al., 2016; Yang and Xiang, 2019). While existing studies have explored attention networks and their role in shooting, the potential impact of ILF-Neurofeedback on this sport remains uncharted territory.

We aim to investigate the effects of ILF-Neurofeedback training on the performance and attentional capabilities of semi-skilled Pistol shooters, using the ANT to measure the impact on attention networks. Our research seeks to contribute valuable insights into the applicability of ILF-Neurofeedback in enhancing performance within the context of shooting—a precision sport where attentional control and mental resilience are paramount. Understanding the potential impact of ILF-Neurofeedback on shooting performance holds practical implications for athletes seeking improved results in this discipline, potentially opening new avenues for optimizing attentional control and overall performance in shooting.

## 2 Materials and methods

### 2.1 Participants

We used G*Power 3.1 to determine the minimal detectable effect (Faul et al., 2007). Consistent with a previous NFT study (Kao et al., 2014), we set the following input parameters for a repeated-measures ANOVA: an alpha value of 0.05, a power of 0.95, a minimum effect size of 0.20, two groups, and two measurements. This computation yielded a required sample size of *N* = 16 for a within-between ANOVA test with two measurements and two groups. Hence, our chosen sample size of 16 was deemed sufficient for the primary objective of our study.

Subsequently, we selected a cohort of 20 pistol shooters from diverse shooting clubs and randomly divided them into two groups: the ILF_NFB group (*N* = 10, consisting of six females and four males, with a mean age of 25.00 ± 8 years) and the control group (*N* = 10, comprising five females and five males, with a mean age of 26.00 ± 5 years). The reason for our use of pistol shooters was due to the availability of the target group and also the relative difficulty of this type of shooting compared to rifle shooting. To meet the semi-skilled criteria, participants were required to have shooting scores within the range of 530 to 550 and to have competed in a maximum of two national competitions. Pistol shooting athletes must register a record in the federation to enter any level. If this record is between 530 and 550, it means that the athlete has fully learned the skill and is ready to enter the competitions and is known as semi-skilled. Athletes with scores of 560 and above are considered skilled and compete at higher levels, while those scoring 570 or 580 and above are recognized as elite shooters at the international level. Additionally, they needed to be free from diagnosed mental illnesses, nervous disorders, mental trauma, or migraines. They should not have medications that might interfere with the testing process. Right-handedness was another inclusion criterion for the shooters.

We obtained ethical approval for all experimental procedures from the Biological Research Ethics Committee of SBU, with the research assigned the Ethics confirmation code IR.SBU.REC.1399.004.

### 2.2 Shooting performance

Shooting performance was assessed using a specialized system known as the Shooter’s Coordination Analysis Target Training (SCATT) system. This system relies on shot results indicators to evaluate the athletes’ performances. It is specifically designed for shooting analysis and comprises two primary components: software (Version 5.28) and hardware. The hardware component includes an optical receiver positioned beneath the pistol, an electronic target that can be situated between 4 and 12 meters from the shooter, and a control unit. Shot results were recorded on a scale ranging from 0 to 10.9. The average results were computed for each shooter (Ball et al., 2003).

Following a comprehensive warm-up, each athlete executed a standard shooting procedure from a distance of 10 meters to the standard target. This involved taking 10 shots in a training setting devoid of pressure. The SCATT system was utilized to assess and determine shooting performance by analyzing the shot results.

### 2.3 Attention network test (ANT)

**Figure 1:**
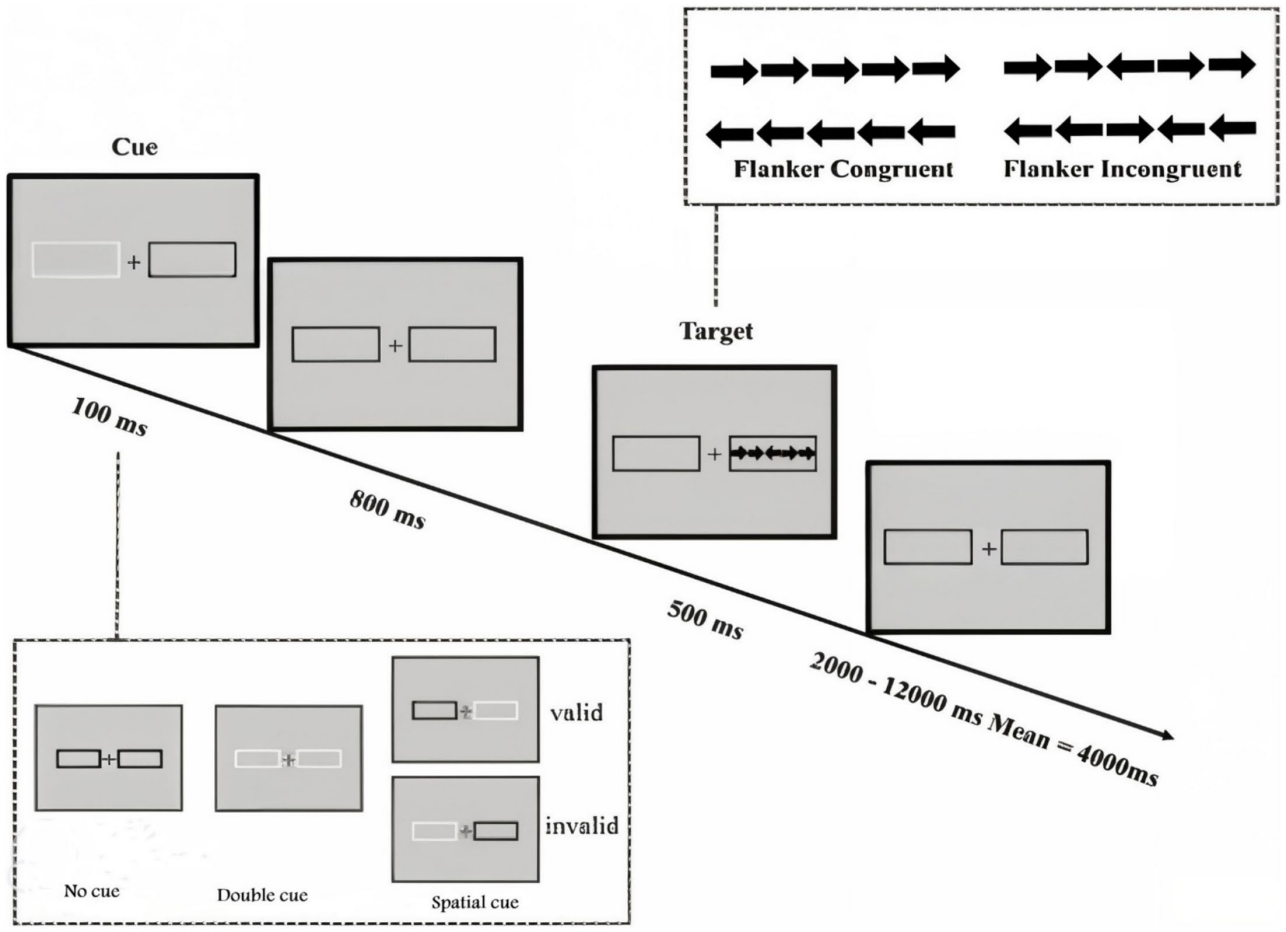
Attention Network Test (ANT) Reprinted from Fan et al. (2009). ANT design and procedure with the four warning cue conditions (no cue, central cue, double cue and spatial cue) and the three flanker conditions (Incongruent, Neutral and Congruent).

Assessment with the Attention Network Test (ANT) utilized the standard 20-min version of the test developed by Fan et al. (2009). The presentation of ANT and data recording utilized the Psych toolbox. The specifics of the ANT task are illustrated in Figure 1.

In this task, each trial initiates with a central fixation point, followed by one of three cue conditions (no-cue, double-cue, spatial-cue). Subsequently, a target display appears 400 milliseconds after the cue offset. The target condition can be congruent or incongruent. Further details regarding cue and target display times, as well as cue-target intervals, are provided in Figure 1. To evaluate participants’ attentional functions, including alerting, orienting, and executive control, an equal number of trials were randomly distributed in all blocks. The test instructions emphasized achieving maximum speed consistent with accuracy. Participants underwent a practice block consisting of 24 full feedback trials. The test phase comprised 288 trials, distributed across three blocks of 96 trials each.

### 2.4 Intervention

#### 2.4.1 ILF neurofeedback

Each member of the intervention group underwent a series of 20 ILF NFB training sessions, each lasting 30 min. These sessions were administered by a certified ILF NFB therapist who had undergone the professional training.

The clinical intervention employed a neurofeedback protocol known as Infra-Low-Frequency Neurofeedback (ILF NFB), developed by Siegfried and Susan Othmer. This approach is described in detail in their works (Othmer, 2011; Othmer and Othmer, 2016). Pre- and post-test assessments using SCATT and ANT were conducted 24 h before the first neurofeedback session and 24 h after the final session. This interval was kept consistent for all participants to minimize variability in performance due to external factors. The neurofeedback training began immediately following this initial assessment, ensuring that the time between assessment and intervention was standardized across participants. During the training, participants were instructed to refrain from additional shooting practice. The same conditions were maintained for both the ILF-NFB and control groups.

#### 2.4.2 Instrumentation and electrode montage

EEG recordings were conducted using a “NeuroAmp II” EEG amplifier with two channels, covering a full band of DC-100 Hz, at a rate of one mega-sample per second, down-sampled to 250 samples per second, and boasting 32-bit resolution. This instrumentation was provided by Corscience GmbH in Germany and operated with the Cygnet© software developed by BEE Medic GmbH in Germany. This system integrates with audio-visually animated feedback (Somatic Vision, USA). It was run on a laptop computer with Windows 10 operating system and connected to an additional high-resolution monitor to display the video animations. All NF sessions took place in an air-conditioned environment where participants could watch the audio-visual video animation “InnerTube” for 30 min per session while sitting comfortably in a chair. During the neurofeedback sessions, real-time feedback was provided using an airplane and tunnel protocol. Participants controlled the movement of an airplane that passed through a tunnel based on their brain activity. Therefore, this animation provides a variable visual indication of the participant’s ongoing neural activity as brain signals change dynamically. The visual feedback was displayed on a high-resolution monitor, and participants were instructed to maintain focus and composure to optimize the airplane’s movement through successive targets in the tunnel. Brain signals control the speed and accuracy of the airplane’s movement. This protocol of ILF training operates on principles of conditioning and reinforcement, guiding participants toward optimal brain states. All visual stimuli were functionally tied to the neurofeedback training. Neurofeedback training integrates two distinct processes: (1) an automated inhibition algorithm targeting classical frequency bands (0.5–40 Hz) to suppress undesired neural activity and (2) feedback derived from slow oscillations (below 0.1 Hz) to promote self-regulation of brain networks. While the inhibition process aligns with classical neurofeedback methodologies, the feedback process represents a broader neuromodulatory approach, potentially fostering self-organization (Othmer and Othmer, 2016; Grin-Yatsenko et al., 2018).

During all neurofeedback sessions, EEG recordings utilized a bipolar derivation in a two-channel montage. Electrode placement followed the 10/20 electrode positioning system recommended by the American Clinical Neurophysiology Society, specifically at locations T3 and T4 (T3-T4), T4 and P4 (T4-P4), and T3 and Fp1 (T3-Fp1) in the 10–20 system. The two-channel montage featured a common reference, Cz, along with a grounding electrode at Fpz. Before attaching the electrodes, the skin in the placement area was cleaned using abrasive paste (Nuprep© from Weaver and Company, USA), and Ten20 Conductive Paste (Weaver and Company, USA) was applied to secure the electrodes and maintain low impedances (<5 kΩ) for all electrodes.

#### 2.4.3 Infra-low frequency neurofeedback

The Cygnet© software processes the EEG and derives the feedback signal. In the ILF-NFB protocol employed in the research, the full-band EEG in the DC-100 Hz range was utilized in the neurofeedback. This process comprised two components, the “ILF training signal” and the “inhibits,” to which specific sets of distinct audio-visual feedback signals were linked. These feedback signals were then presented to participants in the intervention group through computer-generated animations (Grin-Yatsenko et al., 2018; Legarda et al., 2011; Othmer, 2011; Othmer and Othmer, 2016). The channel difference signal informs the ILF training, whereas the sum of channels yields the common-mode signal to inform the EEG-band inhibit scheme.

For the “inhibit” component, the trainee is alerted to supra-threshold excursions within nine adjacent, non-overlapping frequency bands derived from the continuously recorded EEG data within the 0.5–40 Hz frequency spectrum. The threshold values for the nine frequency bands were individually and adaptively adjusted by the Cygnet© software. This adjustment aimed to maintain the individual thresholds at such levels that the inhibits collectively would be engaged no more than 5% of the time. Any sudden large increase in band power within one of these nine frequency bands is capable of triggering the inhibit, which would then become apparent to the trainee through transients in the audio-visual feedback signals.

The ILF “training signal” is derived by way of a low-pass filter customized for each client by the therapist. The resulting signal bandwidth encompasses the range of 0.0001–10 mHz. The ILF signal is dynamically presented within the computer animation using audio-visual feedback signals linked to the “training signal” component through the ILF-NFB software. During the first five ILF-NFB sessions, the therapist identified the optimal frequency settings for each participant based on their individual responses and wellbeing. These personalized settings were then consistently applied throughout the remaining 20 sessions to ensure the effectiveness of the training. In order to optimize ILF NFB training for arousal regulation (Othmer and Othmer, 2016), neurofeedback sessions commenced with a specialist in ILF neurofeedback selecting one of two protocols, namely, (T3-T4) or (T4-P4), based on the completion of a questionnaire assessing the psychological, physiological and mental status of the participants. After five training sessions and the establishment of the optimal training frequency, the ILF NF training proceeded for 20 sessions using three protocols, which changed every 10 min during the 30-min session. This involved placing electrodes at (T4-P4) for the first 10 min, followed by (T4-T3) for the subsequent 10 min, and concluding with (T3-FP1) for the final 10 min.

During the training sessions, individuals sat comfortably in front of a monitor, and electrodes were placed on their scalp. Over the course of 30 min, participants observed a simulated flight through a tunnel, where their brainwave activity controlled the aircraft’s movement through successive targets at the tunnel’s center. Throughout the exercise, participants were required to maintain their composure and alertness, focusing solely on the aircraft, as the speed through the tunnel encoded the ILF signal.

### 2.5 Study design and experimental procedure

**Figure 2:**
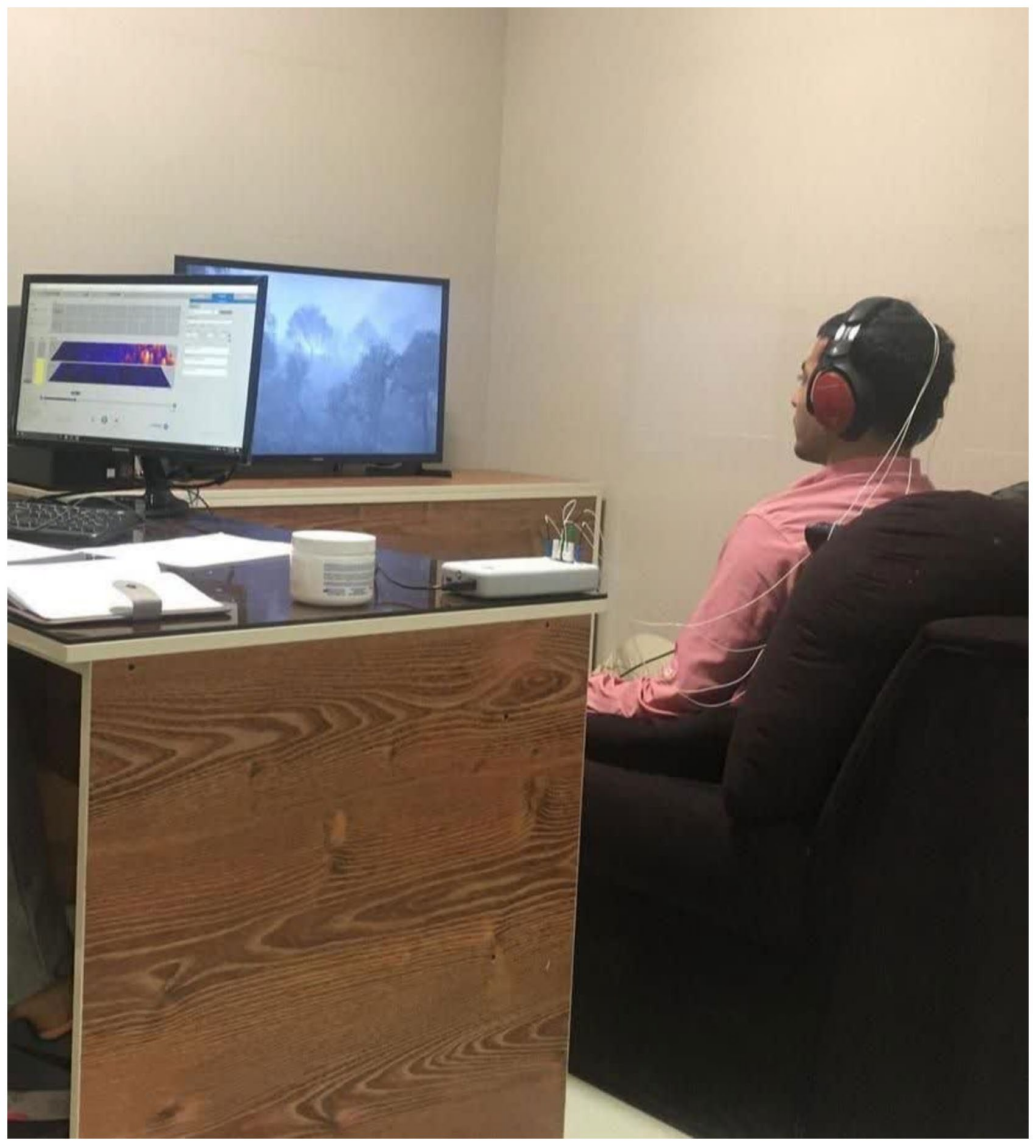
Representation of ILF-neurofeedback setup.

The study follows a pre−/post-test design with both an intervention group and a control group, as illustrated in Figure 2. The participants involved in the study were 20 semi-skilled pistol shooters. Approval for the study was obtained with the Ethics Code IR.SBU.REC.1399.004, and all participants provided voluntary consent before commencing the tests.

Prior to the shooting performance test, participants were instructed to abstain from consuming caffeine and alcohol-containing foods, as well as refraining from playing computer or mobile games during the test. The initial phase involved all participants completing pre-test requirements. During this phase, they were educated on how to conduct the shooting performance test, provided consent forms, and completed personal information questionnaires,

Subsequently, in the test and intervention phase, all participants underwent a general and specialized warm-up for shooting before engaging with the pistol as required. They were then required to shoot 10 shots under pressure in a competitive scenario. Following this, only the 10 participants in the neurofeedback group received ILF neurofeedback sessions lasting 30 min each. The testing in this phase was repeated 10 times, meaning all 20 participants engaged in competitive shooting, while the 10 participants in the intervention group received 20 ILF neurofeedback sessions. Participants in the intervention group started their ILF neurofeedback sessions within a day after completing their initial shooting assessment. The schedule for the 20 neurofeedback sessions was structured to include three sessions per week, typically on non-consecutive days, ensuring consistency and sufficient recovery time between sessions. This aligns with standard neurofeedback protocols, where studies recommend three weekly sessions to optimize training effects while preventing fatigue (Bazzana et al., 2022).

Upon completion of the intervention phase, all participants underwent a post-test phase. This phase included completing ANT and SCATT questionnaires and shooting 10 shots under pressure in a competitive situation.

### 2.6 Statistical analysis

The statistical analysis of the collected data was conducted using SPSS (version 25.0). We conducted a thorough examination of the dataset, assessing skewness and kurtosis to ensure they fell within the specified range of −3 to 3 and to identify any potential outliers (Kline, 2023). To evaluate the interventions’ impact, we employed a 2 × 2 mixed ANOVA for shooting performance and a 2 × 2 × 3 mixed ANOVA for ANT data. Furthermore, the Bonferroni *post hoc* test was used to analyze between-group effects. Within each group, we scrutinized the differences in scores between the pre and post-tests using the paired *t*-test. Furthermore, we determined the effect size using partial eta-squared ( $\eta_{p}^{2}$ ), providing insights into the significance of mean changes over time between the groups. Significance was established at *p* ≤ 0.05, specifically within the group × time interaction context.

## 3 Results

**Figure 3:**
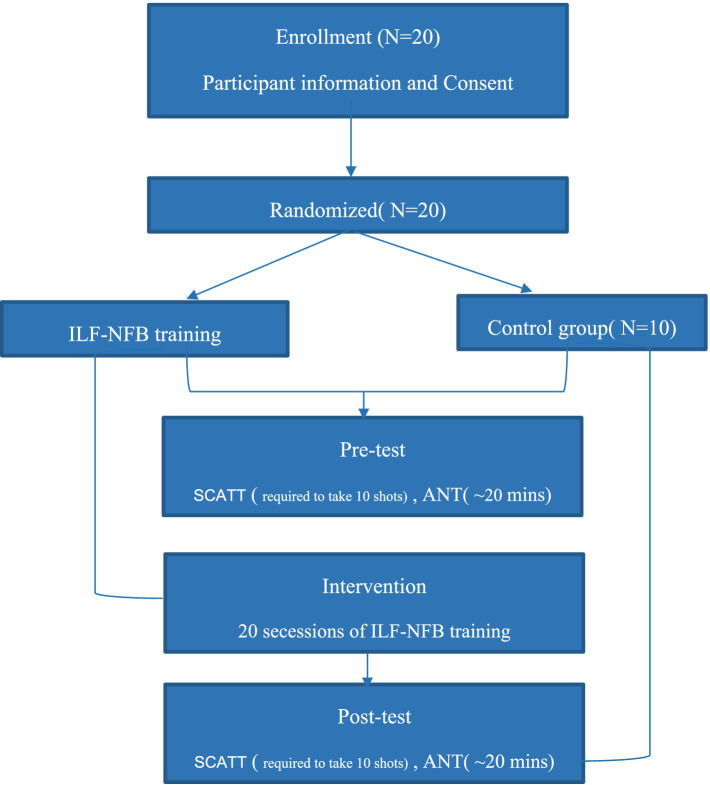
Participant flow through the study. *p = 0.02, **p = 0.001.

Table 1 presents the descriptive statistics of demographic variables, including gender distribution, mean age, years of shooting experience at various competitions, and the range of pistol shooting score. Table 2 and Figure 3 displays the descriptive statistics of the shooting performance score and the attention network test. The efficiency of alerting was calculated using RTs with no cue minus RTs with double cues, the orienting effect is typically assessed by measuring the validity effect, which involves subtracting reaction times (RTs) of valid spatial cue trials from invalid spatial cue trials, and executive function was RTs of incongruent flankers minus RTs of congruent flankers. Additionally, the paired *t*-test results within each group are presented.

**Table 1:** Descriptive statistics of demographic variables.

Group	Biological sex		Age	Shooting experience	Pistol shooting score
	Females	Males			
ILF-NFB	4	4	32.37 ± 3.92	3–4 years	530–550 point
Control	4	4	35 ± 4.85	3–4 years	530–550- point

**Table 2:** Pre and post test score for shotting performance, alerting, orienting and executive function for each group.

Group variables		ILF NFB group		Control group	
		Pre-test (M ± SD)	Post-test (M ± SD)	Pre-test (M ± SD)	Post-test (M ± SD)
Shooting performance		87 ± 7.5	89.96 ± 2.84	93.5 ± 3.2	89.47 ± 22.3
Alerting	RT	37.93 ± 17.64	19.62 ± 9.11	48.31 ± 12.6	49.87 ± 16.81
Orienting	RT	30.17 ± 8.77	22.81 ± 4.66	36.64 ± 11.47	38.96 ± 7.44
Executive function	RT	499 ± 125.64	358.12 ± 44.74	500.62 ± 182.78	648.62 ± 163

Table 2 represents the Mean and Standard Deviation of Attention Networks data in Active and Inactive Groups.

### 3.1 Shooting performance

We used mixed-model ANOVA 2 (groups) × 2 (phase) to analyze the effects of the ILF NFB on shooting performance. Results showed a significant interaction effect of Phase × Group *F*_(1, 14)_ = 11.29, *p* = 0.005, *Partial η*^2^ = 0.44, a significant main effect of Phase *F*_(1, 14)_ = 8.35, *p* = 0.01, *Partial η*^2^ = 0.37 However, the main effect of Group *F*_(1, 14)_ = 0.7, *p* = 0.79, *Partial η*^2^ = 0.005 wasn’t significant, so the main effects were omitted and the interaction was analyzed., so the main effects were omitted, and the interaction was analyzed. We used the Bonferroni *post hoc* test for more analysis in the following. The results are reported in Figure 3.

Therefore, the post-intervention improvement in shooting performance was observed solely in the ILF NFB group, while shooting performance remained unchanged for the pistol shooters in the control group.

### 3.2 Attention network test (ANT)

**Figure 4:**
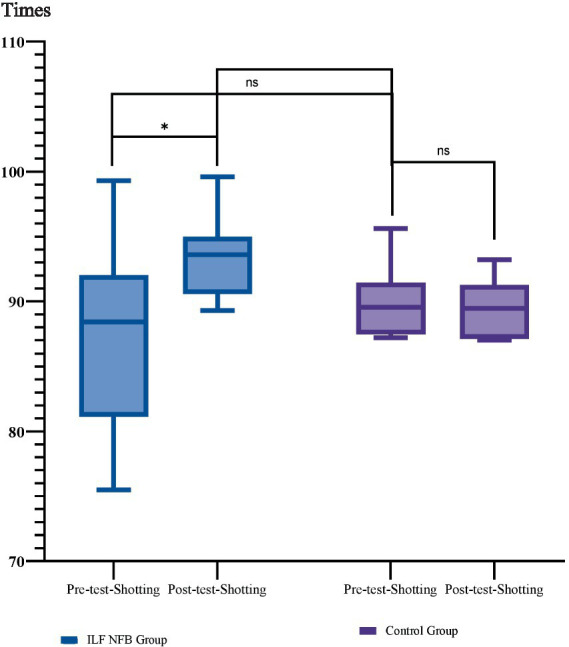
Mean and standard deviation of shooting performance across groups. Group comparisons were analyzed using the Bonferroni *post-hoc* test, while within-group comparisons were assessed using paired *t*-tests. Shooting performance was evaluated using the SCATT testing system Times. *p = 0.02, **p = 0.001, ***p = 0.0001.

**Figure 5:**
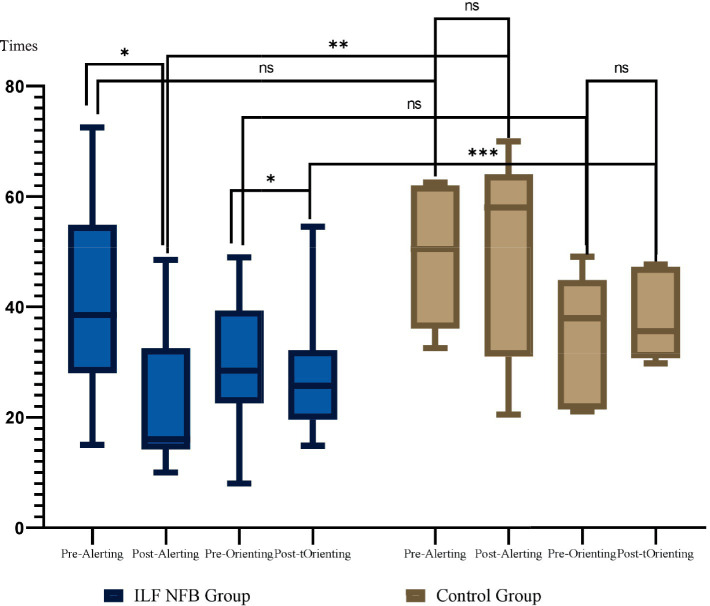
Mean and standard deviation of alerting and orienting across groups. These results are derived from the ANT test. Group comparisons were analyzed using the Bonferroni post hoc test, while within-group comparisons were assessed using paired *t*-tests. Times. *p = 0.002, **p = 0.0001.

We used mixed-model ANOVA 2 (groups) × 2 (phase) × 2 (ANT Variables) to analyze the effects of the ILF NFB on ANT. Results showed a significant main effect of Phase *F*_(1, 14)_ = 431.06, *p* = 0.001, *Partial η*^2^ = 0.96, a significant main effect of Variables *F*_(2, 28)_ = 78.27, *p* = 0.001, *Partial η*^2^ = 0.84, showed a significant main effect of Group *F*_(1, 14)_ = 15.99, *p* = 0.001, *Partial η*^2^ = 0.53, a significant interaction effect of Phase × Group *F*_(1, 14)_ = 7.28, *p* = 0.01, *Partial η*^2^ = 0.34, a significant interaction effect of Group *×* Variables *F*_(2, 28)_ = 6.61, *p* = 0.004, *Partial η*^2^ = 0.32, a significant interaction effect of Phase × Variables *F*_(2, 28)_ = 83.85, *p* = 0.0001, *Partial η*^2^ = 0.85, a significant interaction effect of Phase × Variables × Group *F*_(2, 28)_ = 8.62, *p* = 0.004, *Partial η*^2^ = 0.34. We used the Bonferroni *post hoc* test for more analysis in the following. The results are reported in Figures 4, 5.

**Figure 6:**
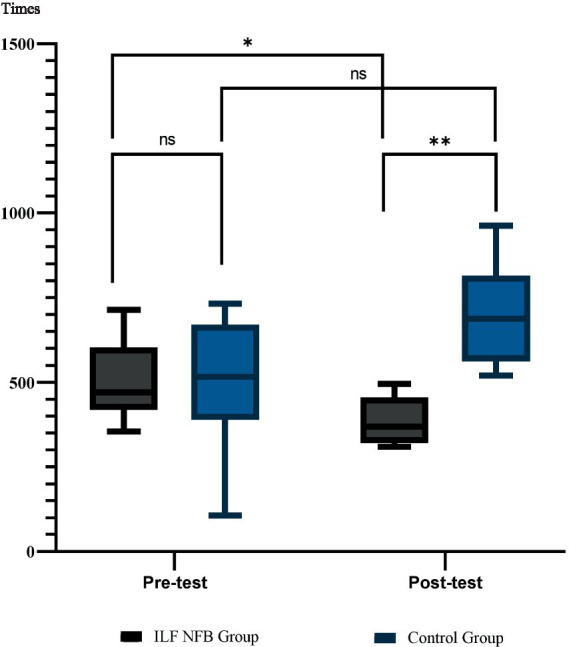
Mean and standard deviation of executive function across groups. These results are derived from the ANT test. Group comparisons were analyzed using the Bonferroni post hoc test, while within-group comparisons were assessed using paired *t*-tests.

The efficiency of alerting was calculated using RTs and error rate with no cue minus RTs with double cues for reaction time and error scores. The paired *t*-test and Bonferroni *post-hoc* revealed that a drop in Alerting RTs can be interpreted as a significant increase in the alerting network of the ILF NFB group. For the control group, no change in alerting RT had occurred pre/post-intervention for the participants in this group (Figure 6).

According to Fan et al. (2009), the orienting effect is typically assessed by measuring the validity effect, which involves subtracting reaction times (RTs) of valid spatial cue trials from invalid spatial cue trials: Orienting Effect = RT (Invalid cues) − RT (Valid cues). The paired *t*-test and Bonferroni *post-hoc* revealed that a significant drop in RTs of orienting was interpreted as a substantial slowdown in the orienting network of the ILF NFB group. In contrast, for the control group, no significant change in orienting RTs had occurred pre- and post-intervention for the participants in this group.

The efficiency of executive function was determined by calculating reaction times (RTs, with congruent cues subtracted from RTs with incongruent cues). The paired *t*-test and Bonferroni post hoc revealed a significant decrease in executive function RTS, reflecting a substantial speedup in the executive function network of the ILF NFB group. In contrast, for the control group, no significant change in executive function RTs had occurred pre- and post-intervention for the participants in this group.

## 4 Discussion

This randomized controlled trial investigates the effect of ILF-Neurofeedback training on shooting performance and attention in semi-skilled players. To our knowledge, our study is a novelty in investigating the effects of ILF-Neurofeedback on semi-skilled Pistol shooters and overcomes specific challenges in providing feedback due to slowly varying signals influencing physiological processes such as arousal, vigilance, and emotional states. Our research into ILF-Rhythms shows a more complex role in how the brain and spinal cord organize and control themselves. This helps us learn a lot more about how ILF-Neurofeedback can improve cognitive and motor skills related to shooting.

The results underscore the distinctive impact of ILF-Neurofeedback on shooting performance, with the ILF-NFB group demonstrating a notable improvement while the control group exhibited minimal change. These findings substantiate the potential efficacy of ILF-Neurofeedback training in enhancing shooting accuracy and overall performance among semi-skilled Pistol shooter.

Our findings in shooting performance align with and contribute to the growing body of literature on neurofeedback training and sports performance (Hammond, 2007; Mirifar et al., 2017; Paul et al., 2012; Rostami et al., 2012; Xiang et al., 2018). Specifically, our results echo positive outcomes observed in studies by Hammond (2007), Paul et al. (2012), and Rostami et al. (2012) in the context of shooting. These studies collectively suggest that neurofeedback, particularly in higher-frequency modalities, has the potential to enhance accuracy, psychological measures, and overall performance in shooting.

The neurofeedback intervention conducted by Landers et al. (1991) closely parallels our study, as both investigations employed distinct approaches—Landers using slow cortical potentials, and our study implementing low infra-frequency neurofeedback. A significant commonality is evident in the shared outcome: both studies demonstrated that neurofeedback training led to an improvement in shooting performance. This convergence in results underscores the potential effectiveness of neurofeedback interventions, highlighting their capacity to enhance skills relevant to shooting despite methodological differences. These findings also lend support to the psychomotor efficiency hypothesis, which suggests that the suppression of task-irrelevant processes and the enhancement of task-relevant processes are associated with superior cognitive-motor processing in the context of expertise (Hatfield and Hillman, 2006).

Comparisons with studies focusing on other neurofeedback modalities reveal shared principles. For instance, the observed improvement in shooting accuracy echoes similar positive outcomes reported in studies utilizing different neurofeedback methods. However, the distinctive nature of ILF-Neurofeedback introduces a novel dimension to these findings, especially considering its impact on Slow Cortical Potentials and its influence on physiological processes like arousal, vigilance, and emotional states. Therefore, it is likely that neurofeedback exercises have been able to improve athletic performance by regulating arousal states. However, further research is needed to fully understand its effects and potential applications in this specific sport.

Neurofeedback exercises have assisted athletes in regulating arousal levels and maintaining the desired level of arousal. According to the theories of Yerkes and Dodson and the hypothesis of optimal arousal, maintaining the desired level of arousal by athletes leads to better performance (Arent and Landers, 2003). These results not only support the psychomotor efficiency hypothesis by revealing the close relationship between the brain cortex and peak sport performance but also prompt researchers to use neurofeedback training (NFT) to improve athlete performance.

As we proceed to the discussion on attention and cognitive aspects, these comparative insights will help contextualize our results within the broader landscape of neurofeedback research in sports performance.

The investigation of attention and cognitive aspects, utilizing the Attention Network Test (ANT), assessed three distinct attention networks—alerting, orienting, and executive function. Remarkably, the ILF-NFB group displayed significant enhancements in all three networks post-intervention, marking this study as a nuanced exploration of ILF-Neurofeedback’s impact on distinct attentional domains.

Participants undergoing ILF-Neurofeedback exhibited faster responses, indicating heightened efficiency across all three attention networks. The improvement in all three attentional networks would, therefore, indicate that the neurofeedback intervention has a broad on the attentional system. This is in line with notions stating that the principal impact of neurofeedback is related to the modulation of the level of arousal, which can then influence multiple components of attention. According to the Yerkes-Dodson Law, an optimum level of arousal should be reached for better cognitive performance, including attentional processes, while too low or too high arousal impairs performance (Yerkes and Dodson, 1908). This effect may be explained by the interconnected nature of attentional networks in the brain. Recent evidence suggests that internal states, such as distractibility and impulsivity, modulate these attentional processes by influencing the neural dynamics of spatial attention and task performance (Amengual et al., 2022). For example, improvements in arousal regulation through neurofeedback can enhance baseline alertness, which, in turn, may facilitate more efficient orienting and conflict-resolution processes within the attentional system (Fan et al., 2009; Sadaghiani and D’Esposito, 2015).

Alerting network: Following ILF-Neurofeedback training, the ILF-NFB group exhibited a significant enhancement in the alerting network. This improvement suggests that post-ILF-Neurofeedback, participants can efficiently attain and sustain a heightened level of vigilance during tasks, implying improved vigilance and activation levels. This positive development aligns with broader neurofeedback literature, emphasizing the modifiability of alertness levels through focused interventions.

Orienting network: Post-intervention, the ILF-NFB group showed a significant positive impact on the orienting network. This result supports the idea that ILF-Neurofeedback enhances mechanisms related to spatial orientation, crucial for precision in shooting. The observed improvement suggests that participants undergoing ILF-Neurofeedback exhibit enhanced selective attention, contributing to improved pistol shooting performance. If ILF induced greater activation of the orienting attentional system, it would be expected that spatial attention reaction times on valid trials would increase, leading to a longer delay in reallocation of attention during invalid trials. However, the reaction times of the orienting attentional system were reduced. In other words, there was an overall improvement in performance that did not depend on whether the cue was valid or invalid. Therefore, the reduction in the RT difference post-treatment suggests that the treatment does not intensify spatial attention allocation but minimizes the cost of reallocating attention when cues are invalid (Bucker and Theeuwes, 2014). It seems that ILF training improves efficiency in orienting and reorienting spatial attention. Our result is consistent with Engelmann and Pessoa’s (2007) findings. ILF training did not interact with cue validity, indicating that ILF generally affected performance instead of improving attentional orienting. An improvement in performance due to ILF training that does not interact with cue validity may indicate a general improvement due to the optimal level of arousal.

Executive function network: Similarly, the executive function network displayed a significant improvement in the ILF-NFB group, highlighted by shorter response times and reduced errors. This underscores ILF-Neurofeedback’s potential in enhancing higher-order cognitive processes crucial for pistol shooting performance.

Importantly, the concept of self-regulation is inherent in both mindfulness and neurofeedback practices (Crivelli et al., 2019; Ford et al., 2016). Our findings align with this, suggesting that practice, particularly mindfulness meditation, can enhance attention network efficiency (Kwak et al., 2020).

Our results are consistent with a body of research indicating that neurofeedback training can enhance attention networks or cognitive performance in athletes, leading to improved overall performance (Barnea et al., 2004; Brito et al., 2022; Mikicin, 2015; Xiang et al., 2018). Comparisons with existing literature highlight the unique contribution of ILF-Neurofeedback, addressing individual attention networks and adding granularity to the understanding of its impact on sports performance.

Theoretical frameworks, such as the Yerkes-Dodson Law and the Optimal Arousal Hypothesis, support the idea that maintaining an optimal level of arousal enhances cognitive functions (Arent and Landers, 2003). Our results align with these theories, suggesting that ILF-Neurofeedback aids athletes in regulating arousal levels, contributing to improved attention and cognitive performance.

## 5 Conclusion

In summary, our research not only deepens the understanding of ILF-Neurofeedback’s influence on shooting performance but also extends its benefits to attention and cognitive functions. These findings contribute valuable insights to the broader discourse on neurofeedback interventions in sports performance, benefitting both researchers and practitioners. Aligning with the psychomotor efficiency hypothesis, our results suggest that the development of motor expertise through ILF-Neurofeedback corresponds to physiological enhancements, emphasizing reduced interference and increased task-related cortical processing as pivotal elements for optimal performance. However, a cautious interpretation is essential, given certain limitations in our study. While illuminating the positive effects of ILF-Neurofeedback on attention and cognitive aspects, it is crucial to recognize the need for further research into underlying mechanisms. Additionally, investigating the transferability of these cognitive benefits to real-world shooting scenarios presents an intriguing avenue for future studies, particularly considering shooting’s closed skill nature. A limitation is the use of passive control groups; future studies incorporating a sham group would undoubtedly be welcomed. However, it can be argued that the training process is inherently sham-controlled because it is covert, at least with respect to the ILF component.

## Data Availability

The raw data supporting the conclusions of this article will be made available by the authors, without undue reservation.
